# Analysis of Train–Track–Bridge Coupling Vibration Characteristics for Heavy-Haul Railway Based on Virtual Work Principle

**DOI:** 10.3390/s23208550

**Published:** 2023-10-18

**Authors:** Nanhao Wu, Hongyin Yang, Haleem Afsar, Bo Wang, Jianfeng Fan

**Affiliations:** 1 School of Civil Engineering and Architecture, Wuhan Institute of Technology, Wuhan 430073, China; woshiwunanhao123@163.com; 2 National Key Laboratory of Bridge Intelligent and Green Construction, Wuhan 430034, China; wangbohust@126.com; 3 Department of Civil and Airport Engineering, College of Civil Aviation, Nanjing University of Aeronautics and Astronautics, Nanjing 210016, China; afsarhaleem@nuaa.edu.cn; 4 Wuhan Mafangshan Engineering Structure Testing Co., Ltd., Wuhan 430070, China; yanhun25@163.com

**Keywords:** heavy-haul railway bridge, train–track–bridge coupling system, virtual work principle, track irregularity, displacement, acceleration

## Abstract

This paper introduces an innovative model for heavy-haul train–track–bridge interaction, utilizing a coupling matrix representation based on the virtual work principle. This model establishes the relationship between the wheel–rail contact surface and the bridge–rail interface concerning internal forces and geometric constraints. In this coupled system’s motion equation, the degrees of freedom (DOFs) of the wheelsets in a heavy-haul train lacking primary suspension are interdependent. Additionally, the vertical and nodding DOFs of the bogie frame are linked with the rail element. A practical application, a Yellow River Bridge with a heavy-haul railway line, is used to examine the accuracy of the proposed model with regard to discrepancy between the simulated and measured displacement ranging from 1% to 11%. A comprehensive parametric analysis is conducted, exploring the impacts of track irregularities of varying wavelengths, axle load lifting, and the degradation of bridge stiffness and damping on the dynamic responses of the coupled system. The results reveal that the bridge’s dynamic responses are particularly sensitive to track irregularities within the wavelength range of 1 to 20 m, especially those within 1 to 10 m. The vertical displacement of the bridge demonstrates a nearly linear increase with heavier axle loads of the heavy-haul trains and the reduction in bridge stiffness. However, there is no significant rise in vertical acceleration under these conditions.

## 1. Introduction

With the growing demand for lot cargo transport, the operation of heavier, longer and faster trains must achieve a harmonious balance between carrying capacity and efficiency [1,2]. The planned heavy-haul railway line is disrupted by topographical obstacles such as rugged mountains, deep valleys, rivers, and other existing railway lines. The construction of bridges plays a crucial role in ensuring the uninterrupted operation of the railway line. The heavy and cyclic axle loads from trains exert high-intensity and short-time dynamic impacts on both the track and the bridge. Much attention is paid to the analysis of train–track–bridge coupling vibration characteristics caused by the dynamic impacts of heavy-haul trains [3].

Heavy-haul trains involve the significant amplitude and frequency of loading cycles, resulting in the excessive deformation and degradation of tracks and bridges [4,5,6]. The track system, consisting of rails, sleepers, and ballast, serves as a conduit for transferring the dynamic load of heavy-haul trains to the bridge deck. At the same time, the vibration of bridges and tracks exerts an adverse impact on running trains, thereby compromising the stability and safety of train operation [7]. The dynamic responses of the heavy-haul train, track, and bridge subsystems can be obtained through the establishment of a train–track–bridge coupling system. Therefore, the establishment of a train–track–bridge coupling system for heavy-haul railways holds immense significance in terms of the design, operation, and maintenance of railway bridges.

So far, numerical simulation has evolved from the simplest moving load model to the train–bridge coupling model, excluding the track subsystem, and ultimately to the refined train–track–bridge coupling model [8,9,10,11]. The wheel–rail contacts and the rail–bridge interaction play an essential role in determining the dynamic responses of trains, tracks, and bridges. In the iterative method, the subsystems of train and track are coupled through the balance of forces and the compatibility of displacement acting on the wheel–rail contact point [12]. Wang et al. [13] presented an iterative approach for solving the equations of motion pertaining to the vehicle and the track, respectively. The iteration persists until the discrepancy in the interaction force between the wheel and the rail falls below the specified tolerance. Zhang et al. [14] developed an intersystem iterative model, which solves the vehicle and bridge motions throughout the entire simulation. The updated dynamic responses form a new excitation to interact with each other’s system until the specified error threshold is reached. Another way to solve the dynamic responses in the time domain is to consider the train–track–bridge as a globally interconnected large-scale system for coupling [15,16]. Lou et al. [17] regarded the wheel–rail contact force as the internal force, and utilized the principle of total potential energy to derive the motion equation for the train–track–bridge system. Chen et al. [18] approached the vehicle and substructure as a holistic system, incorporating interconnected matrices of stiffness, damping, and mass through the energy variational principle and wheel–rail contact geometry. Compared with the aforementioned two methods, the iterative process of wheel–rail force may not easily or slowly converge due to the drastic dynamic contact changes between wheelsets and rails for heavy-haul trains. The coupling method employs a step-by-step integration method to obtain the dynamic responses of the train, track and bridge simultaneously, which avoids the numerical diffusion problem in the iterative process.

The majority of the relevant numerical models used in the existing literature are derived and analyzed for high-speed railway bridges and high-speed trains [19,20,21]. The main concern in high-speed railway bridge design is the severe vibration caused by vehicle–bridge resonance [22,23]. However, the vibration source complexity of heavy-haul railways surpasses that of high-speed railways. The vibration of heavy-haul railway bridges is primarily attributed to factors such as high axle loads [24], diverse car body suspension modes [25], serious rail wear [26], and ballast softening [27]. The static axle load of high-speed trains typically does not exceed 170 kN, while for heavy-haul trains it may range from 250 kN to 350 kN. The increase in train loading and train formation is likely to have a significant impact on substructures, resulting in dynamic amplifications, fatigue damage, and ballast settlement. Shi et al. [24] established a type of bridge–embankment transition model to predict the acceleration of the transition zone below a heavy-haul railway line under the existing loads. Wang et al. [26] investigated the competition and constrained relationship between fatigue crack damage and side wear for a heavy-haul railway rail. Feng et al. [28] evaluated the mechanical properties and weld fatigue behavior of orthotropic steel bridge decks in heavy-haul railway bridges. Emrah et al. [29] described a detailed investigation of the dynamic behavior of heavy-haul railway bridges through a moving load and moving mass model under various parameter variables, including bridge span, normalized train length, normalized train mass, bridge deck stiffness and train speed. In summary, the dynamic responses of heavy-haul railways need to be obtained through a comprehensive coupling analysis of train–track–bridge systems. However, there is currently a dearth of research in the field of heavy-haul railway bridges.

This study aims to establish and validate an innovative coupling model for heavy-haul trains, tracks, and bridges. This model accurately simulates the dynamic behavior of complex heavy-haul trains, considering that most heavy-haul trains lack primary suspension, unlike high-speed trains. In these heavy-haul trains, the bogie is connected to the rail element through vertical and nodding motions, necessitating the derivation of the wheelsets’ motion from the bogie frame. Utilizing the principle of virtual work, motion equations for each subsystem are derived. Notably, this approach avoids the use of wheel–rail and bridge–rail interfaces as system boundaries, eliminating the need for iterative solutions. The model’s validity is confirmed through its application to a continuous rigid frame bridge with a heavy-haul railway line. Furthermore, the study includes a parametric analysis, considering track irregularities of varying wavelengths, axle load variations, and the degradation of bridge stiffness and damping, thus comprehensively evaluating the system’s dynamic responses.

## 2. Modeling of Heavy-Haul Train–Track–Bridge Coupling System

### 2.1. Models of Vehicle, Rail and Bridge

**Figure 1:**
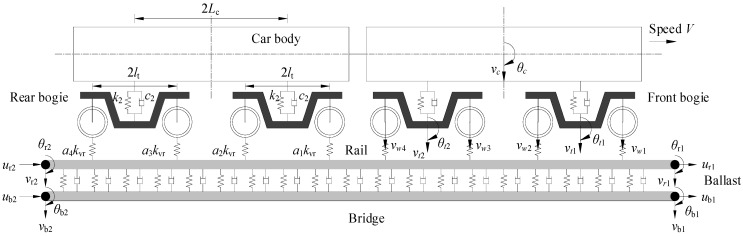
Heavy-haul train–track–bridge coupling model.

The dynamic characteristics in the axial and vertical directions are exclusively considered in this study, as they are the primary factors contributing to bridge vibration caused by heavy-haul trains, while disregarding transverse deformation. A typical heavy-haul train–track–bridge coupling model is schematically shown in Figure 1.

This model is constructed based on the following five assumptions:

(1) Rails and bridges exhibit elastic, homogeneous and isotropic behaviors, which are simulated by plane Euler–Bernoulli beam;
(2) The wheel–rail contact region is a small elliptical area, and the linear Hertz contact theory is employed to simulate the wheel–rail contact relationship;
(3) The heavy-haul train is modeled as a four-axle multi-rigid-body system;
(4) The heavy-haul trains maintain a consistent velocity along the bridge span direction, regardless of the longitudinal connection and vibration between the car bodies;
(5) Ballast is modeled as a continuous spring and damping system to provide track support, and the left and right rails are merged into a single strand in the model.

### 2.2. Equation of Motion for the Bridge by Virtual Work Principle

Firstly, it is assumed that the length of the bridge element is denoted by *l*_b_, and the Young’s modulus and density per unit length of the bridge are denoted by *E*_b_ and *ρ*_b_, respectively. The symbols *k*_rb_ and *c*_rb_ denote the spring stiffness and damping coefficients of the railway ballast, respectively. The bridge element is characterized by three degrees of freedom (DOFs) at each node, namely, a longitudinal displacement *u*_b_, a vertical displacement *v*_b_ and a rotation *θ*_b_ about an axis normal to the plane of paper. 

**Figure 2:**
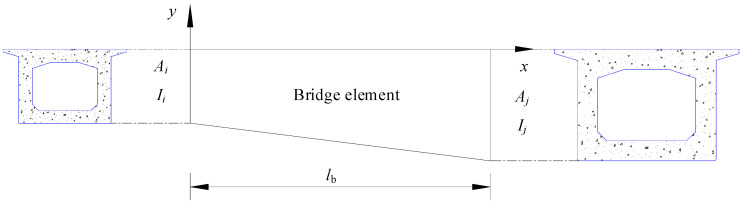
The beam element of the linear variable cross-section.

Assuming that the cross-section of the bridge element is variable, the interpolation method can be employed to express the cross-sectional area and moment of inertia between the nodes based on the parameters of the section at both ends of the element. The cross-section area and moment of inertia at the coordinate *x* are denoted by *A*(*x*) and *I*(*x*), respectively. The beam element of the linear variable’s cross-section is shown in Figure 2.

In the next section, the equation of motion for the bridge is derived by considering the virtual work contributions of the inertia force, elastic deformation, interaction between the rail and bridge, as well as external forces. The virtual work of a bridge element with variable cross-section can be expressed by:

(1) $$\begin{array}{l} \int_{0}^{l_{b}}E_{b}A_{b}(x)u_{b}^{\prime }\delta u_{b}^{\prime }dx+\int_{0}^{l_{b}}E_{b}I_{b}(x)v_{b}^{\prime \prime }\delta v_{b}^{\prime \prime }dx+\int_{0}^{l_{b}}\rho_{b}A_{b}(x){\ddot{u}}_{b}\delta u_{b}dx+\int_{0}^{l_{b}}\rho_{b}A_{b}(x){\ddot{v}}_{b}\delta v_{b}dx+\\ \int_{0}^{l_{b}}c_{b}{\dot{u}}_{b}\delta u_{b}dx+\int_{0}^{l_{b}}c_{b}{\dot{v}}_{b}\delta v_{b}dx+\int_{0}^{l_{b}}k_{\mathrm{rb}}\left(v_{b}-v_{r}\right)\delta v_{b}dx+\int_{0}^{l_{b}}c_{\mathrm{rb}}\left({\dot{v}}_{b}-{\dot{v}}_{r}\right)\delta v_{b}dx=\delta d_{b}f_{b}^{T} \end{array}$$

with

$$\;A_{b}(x)=A_{i}+\frac{A_{j}-A_{i}}{l_{b}}x\;I_{b}(x)=I_{i}+\frac{I_{j}-I_{i}}{l_{b}}x\;\;\;\;\;\;\;d_{b}=\left\{\begin{array}{cccccc} u_{b1} & v_{b1} & \theta_{b1} & u_{b2} & v_{b2} & \theta_{b2} \end{array}\right\}$$

where $d_{b}$ is the node motion vector at both ends of the bridge element; $f_{b}$ is external loading force vector acting on the bridge element; the superscripts “⋅” and “⋅⋅” are the first and second derivatives with respect to time, and the superscripts “′” and “″” are the first and second derivatives with respect to coordinates.

### 2.3. Equation of Motion for the Rail by Virtual Work Principle

Firstly, it is assumed that the length of the rail element is denoted by *l*_r_, while the Young’s modulus and density per unit length of the rail are denoted by *E*_r_ and *ρ*_r_, respectively. The symbols *A*_r_ and *I*_r_ denote the cross-section area and moment of inertia of the rail, respectively. The rail element is characterized by three DOFs at each node, namely, a longitudinal displacement *u*_r_, a vertical displacement *v*_r_ and a rotation *θ*_r_ about an axis normal to the plane of paper.

The geometric compatibility condition of the relative vertical displacement between the wheel–rail contact at the *k*th wheelset and the *t*th moment can be expressed by:

(2) $$y_{k}^{s}=v_{wk}(t)-v_{r}(x_{k},t)-IR_{y}(x_{k})$$

where $v_{wk}(t)$ is the absolute vertical displacement of the *k*th wheelset at the *t*th moment; $v_{r}$ is the absolute vertical displacement of the rail at the contact point $x_{k}$ and the moment *t*; $IR_{y}$ is the vertical geometric irregularity of the rail at the contact point $x_{k}$.

Considering that there is no tension in the wheel–rail contact, it is unnecessary to model wheel–rail contact and separation separately. Based on the assumption (2), the linear elastic stiffness coefficient of wheel–rail Hertz contact is expressed by $a_{wk}k_{\mathrm{vr}}$. The contact coefficient $a_{wk}$ should adhere to the following relationship:

(3) $$\left\{\begin{array}{c} \begin{array}{c} a_{wk}=1\;y_{k}^{s}>0\\ a_{wk}=0\;y_{k}^{s}<0 \end{array} \end{array}\right.$$

where the subscript “w*k*” denotes the *k*th wheelset.

In the next section, the equation of motion for the rail is derived by considering the virtual work contributions of the inertia force, the elastic deformation, the interaction between the rail and bridge, the contact force between the rail and wheelset, as well as external forces. The virtual work of the rail element can be expressed by:

(4) $$\begin{array}{l} \int_{0}^{l_{r}}E_{r}A_{r}u_{r}^{\prime }\delta u_{r}^{\prime }dx+\int_{0}^{l_{r}}E_{r}I_{r}v_{r}^{\prime \prime }\delta v_{r}^{\prime \prime }dx+\int_{0}^{l_{r}}\rho_{r}A_{r}{\ddot{u}}_{r}\delta u_{r}dx+\int_{0}^{l_{r}}\rho_{r}A_{r}{\ddot{v}}_{r}\delta v_{r}dx+\\ \int_{0}^{l_{r}}k_{\mathrm{rb}}\left(v_{r}-v_{b}\right)\delta v_{r}dx+\int_{0}^{l_{r}}c_{\mathrm{rb}}\left({\dot{v}}_{r}-{\dot{v}}_{b}\right)\delta v_{r}dx+\sum_{k=0}^{n_{w}}a_{wk}k_{\mathrm{wr}}\left(v_{r}-v_{wk}-IR_{y}\right)\delta v_{r}=\delta d_{r}f_{r}^{T} \end{array}$$

with

$$d_{r}=\left\{\begin{array}{cccccc} u_{r1} & v_{r1} & \theta_{r1} & u_{r2} & v_{r2} & \theta_{r2} \end{array}\right\}$$

where $d_{r}$ is the node motion vector at both ends of the rail element; $f_{r}$ is the external loading force vector acting on the rail element; $n_{w}$ is the number of wheelsets on the rail element.

### 2.4. Equation of Motions for a Heavy-Haul Train by Virtual Work Principle

The heavy-haul train simulation employs a multi-rigid-body dynamic model, which consists of a car body, a front bogie, a rear bogie, a secondary suspension, and four wheelsets. The car body and the bogie frames of the *i*th heavy-haul railway vehicle possess two DOFs, respectively, namely, vertical motion ($v_{c}$, $v_{t1}$ and $v_{t2}$) and nodding motion ($\theta_{c}$, $\theta_{t1}$, and $\theta_{t2}$), where the subscript “c” denotes car body, “t1” denotes front bogie, and “t2” denotes rear bogie. Since there is no primary suspension in the majority of heavy-haul trains, the DOFs of the wheelsets are no longer independent and can be derived from the motion of bogie. The positive direction is defined as downward for vertical motion and clockwise for nodding motion. The wheelset is coupled with the rail element through the Hertz contact spring.

The equation of motion for the car body is derived by considering the virtual work contributions of the inertia force acting on the car body, the interaction between the car body and the bogies, as well as gravity. The virtual work of the car body can be expressed by:

(5) $$\begin{array}{l} m_{c}{\ddot{v}}_{c}\delta v_{c}+I_{c}{\ddot{\theta }}_{c}\delta \theta_{c}+k_{2}(v_{c}-v_{t1})\delta v_{c}+k_{2}(v_{c}-v_{t2})\delta v_{c}+k_{2}L_{c}(L_{c}\theta_{c}-v_{t1})\delta \theta_{c}+\\ k_{2}L_{c}(L_{c}\theta_{c}+v_{t2})\delta \theta_{c}+c_{2}({\dot{v}}_{c}-{\dot{v}}_{t1})\delta v_{c}+c_{2}({\dot{v}}_{c}-{\dot{v}}_{t2})\delta v_{c}+c_{2}L_{c}(L_{c}{\dot{\theta }}_{c}-{\dot{v}}_{t1})\delta \theta_{c}+\\ c_{2}L_{c}(L_{c}{\dot{\theta }}_{c}+{\dot{v}}_{t2})\delta \theta_{c}=m_{c}g\delta v_{c} \end{array}$$

where $m_{c}$ and $I_{c}$ are the mass and rotational inertia of the car body; $k_{2}$ and $c_{2}$ are the spring stiffness and damping coefficient of secondary suspension; $L_{c}$ is half of the longitudinal distance between the center of gravity of the front bogie and the rear bogie.

The equation of motion for the front bogie is derived by considering the virtual work contributions of the inertia force acting on the front bogie, the interaction between the car body and the front bogie, the contact between the wheelsets and the rail, as well as gravity. The virtual work of the front bogie can be expressed by:

(6) $$\begin{array}{l} (m_{t}+2m_{w}){\ddot{v}}_{t1}\delta v_{t1}+(I_{t}{\ddot{\theta }}_{t1}+2m_{w}l_{t}^{2})\theta_{t1}\delta \theta_{t1}+k_{2}(v_{t1}-v_{c}-L_{c}\theta_{c})\delta v_{t1}+c_{2}({\dot{v}}_{t1}-{\dot{v}}_{c}-L_{c}{\dot{\theta }}_{c})\delta v_{t1}+\\ a_{w1}k_{\mathrm{wr}}(v_{t1}+l_{t}\theta_{t1}-v_{r}-IR_{y1})\delta v_{t1}+a_{w1}k_{\mathrm{wr}}l_{t}(v_{t1}+l_{t}\theta_{t1}-v_{r}-IR_{y1})\delta \theta_{t1}+a_{w2}k_{\mathrm{wr}}(v_{t1}-l_{t}\theta_{t1}-v_{r}-IR_{y2})\delta v_{t1}-\\ a_{w2}k_{\mathrm{wr}}l_{t}(v_{t1}-l_{t}\theta_{t1}-v_{r}-IR_{y2})\delta \theta_{t1}=(m_{t}+2m_{w})g\delta v_{t1} \end{array}$$

where $m_{t}$ and $I_{t}$ are the mass and rotational inertia of the bogie frame; $m_{w}$ is the mass of the wheelset; $l_{t}$ is half of the bogie axle’s base; the subscripts “w1” and “w2” are the front and rear wheelsets of the front bogie.

The equation of motion for the rear bogie is derived by considering the virtual work contributions of the inertia force acting on the rear bogie, the interaction between the car body and the rear bogie, the contact between the wheelsets and the rail, as well as gravity. The virtual work of the rear bogie can be expressed by:

(7) $$\begin{array}{l} (m_{t}+2m_{w}){\ddot{v}}_{t2}\delta v_{t2}+(I_{t}{\ddot{\theta }}_{t2}+2m_{w}l_{t}^{2})\theta_{t2}\delta \theta_{t2}+k_{2}(v_{t2}-v_{c}+L_{c}\theta_{c})\delta v_{t2}+c_{2}({\dot{v}}_{t2}-{\dot{v}}_{c}+L_{c}{\dot{\theta }}_{c})\delta v_{t2}+\\ a_{w3}k_{\mathrm{wr}}(v_{t2}+l_{t}\theta_{t2}-v_{r}-IR_{y3})\delta v_{t2}+a_{w3}k_{\mathrm{wr}}l_{t}(v_{t2}+l_{t}\theta_{t2}-v_{r}-IR_{y3})\delta \theta_{t2}+a_{w4}k_{\mathrm{wr}}(v_{t2}-l_{t}\theta_{t2}-v_{r}-IR_{y4})\delta v_{t2}-\\ a_{w4}k_{\mathrm{wr}}l_{t}(v_{t2}-l_{t}\theta_{t2}-v_{r}-IR_{y4})\delta \theta_{t2}=(m_{t}+2m_{w})g\delta v_{t2} \end{array}$$

where the subscripts “w3” and “w4” are the front and rear wheelsets of the rear bogie.

The equations of motion for wheelsets are not independent. The displacements, velocities, and accelerations of the wheelset are dependent on the corresponding displacements, velocities, and accelerations of the bogie.

(8) $$v_{w1}=v_{t1}+l_{t}\theta_{t1},\;{\dot{v}}_{w1}={\dot{v}}_{t1}+l_{t}{\dot{\theta }}_{t1},\;{\ddot{v}}_{w1}={\ddot{v}}_{t1}+l_{t}{\ddot{\theta }}_{t1}$$

(9) $$v_{w2}=v_{t1}-l_{t}\theta_{t1},\;{\dot{v}}_{w2}={\dot{v}}_{t1}-l_{t}{\dot{\theta }}_{t1},\;{\ddot{v}}_{w2}={\ddot{v}}_{t1}-l_{t}{\ddot{\theta }}_{t1}$$

(10) $$v_{w3}=v_{t2}+l_{t}\theta_{t2},\;{\dot{v}}_{w3}={\dot{v}}_{t2}+l_{t}{\dot{\theta }}_{t2},\;{\ddot{v}}_{w3}={\ddot{v}}_{t2}+l_{t}{\ddot{\theta }}_{t2}$$

(11) $$v_{w4}=v_{t2}-l_{t}\theta_{t2},\;{\dot{v}}_{w4}={\dot{v}}_{t2}-l_{t}{\dot{\theta }}_{t2},\;{\ddot{v}}_{w4}={\ddot{v}}_{t2}-l_{t}{\ddot{\theta }}_{t2}$$

## 3. Assembly and Solution of Heavy-Haul Train–Track–Bridge Coupling Matrix

The heavy-haul train, track and bridge constitute an integrated system in which the vibration characteristics vary with the passage of time during train crossings on the railway. Based on the equation of motion derived from the virtual work principle in Section 2 and following the rule of “seat by number” for matrix formation, the dynamic equations of motion for the heavy-haul train–track–bridge coupling system can be expressed in matrix form, that is:

(12) $$\left[\begin{array}{ccc} M_{\mathrm{bb}} & 0 & 0\\ 0 & M_{\mathrm{rr}} & 0\\ 0 & 0 & M_{\mathrm{vv}} \end{array}\right]\left\{\begin{array}{c} {\ddot{X}}_{b}\\ {\ddot{X}}_{r}\\ {\ddot{X}}_{v} \end{array}\right\}+\left[\begin{array}{ccc} C_{\mathrm{bb}} & C_{\mathrm{br}} & 0\\ C_{\mathrm{rb}} & C_{\mathrm{rr}} & 0\\ 0 & 0 & C_{\mathrm{vv}} \end{array}\right]\left\{\begin{array}{c} {\dot{X}}_{b}\\ {\dot{X}}_{r}\\ {\dot{X}}_{v} \end{array}\right\}+\left[\begin{array}{ccc} K_{\mathrm{bb}} & K_{\mathrm{br}} & 0\\ K_{\mathrm{rb}} & K_{\mathrm{rr}} & K_{\mathrm{rv}}\\ 0 & K_{\mathrm{vr}} & K_{\mathrm{vv}} \end{array}\right]\left\{\begin{array}{c} X_{b}\\ X_{r}\\ X_{v} \end{array}\right\}=\left\{\begin{array}{c} F_{b}\\ F_{r}\\ F_{v} \end{array}\right\}$$

where **M**, **C** and **K** are the submatrices of mass, damping and stiffness respectively; X, $\dot{X}$ and $\ddot{X}$ are the vectors of displacement, velocity and acceleration, respectively; **F** is the vector of force; the subscripts “b”, “r” and “v” are the bridge, rail and heavy-haul vehicle, respectively.

### 3.1. Submatrices of Bridge and Bridge-Rail Coupling

The symbols “$N_{u,j}$” and “$N_{v,j}$” represent axial and vertical interpolation functions of the bridge element with the same order 1 × 6, respectively. The former is the primary Hermitian interpolation function and the latter is the cubic Hermitian interpolation function [30], namely,

(13) $$v_{w4}=v_{t2}-l_{t}\theta_{t2},\;{\dot{v}}_{w4}={\dot{v}}_{t2}-l_{t}{\dot{\theta }}_{t2},\;{\ddot{v}}_{w4}={\ddot{v}}_{t2}-l_{t}{\ddot{\theta }}_{t2}$$

(14) $$N_{u,j}=\left\{\begin{array}{cccccc} N_{1} & 0 & 0 & N_{2} & 0 & 0 \end{array}\right\}$$

with

$$\begin{array}{l} N_{1}=1-\left(x/l_{j}\right),\;N_{2}=x/l_{j},\;N_{3}=1-3{\left(x/l_{j}\right)}^{2}+2{\left(x/l_{j}\right)}^{3}\\ N_{4}=x{\left(1-x/l_{j}\right)}^{2},\;N_{5}=3{\left(x/l_{j}\right)}^{2}-2{\left(x/l_{j}\right)}^{3},\;N_{6}=x\left[{\left(x/l_{j}\right)}^{2}-x/l_{j}\right]; \end{array}$$

where *x* is the horizontal distance from the left node of the element; $l_{j}(j=b,r)$ is the length of the beam element.

The axial and vertical displacements of the bridge element related to the nodal DOFs can be respectively expressed by:

(15) $$v_{b}\left(x\right)=N_{u,b}d_{b}^{T}$$

(16) $$u_{b}\left(x\right)=N_{v,b}d_{b}^{T}$$

The axial and vertical displacements of the rail element related to the nodal DOFs can be respectively expressed by:

(17) $$v_{r}\left(x\right)=N_{u,r}d_{r}^{T}$$

(18) $$u_{r}\left(x\right)=N_{v,r}d_{r}^{T}$$

Substituting the displacement fields, i.e., Equations (15)–(18), into the virtual work Equation (1) yields the bridge and bridge–rail coupling submatrices. The bridge submatrix is marked with the subscript “bb”. The mass submatrix $M_{\mathrm{bb}}$ of the bridge with the order $n_{b}\times n_{b}$ can be expressed by:

(19) $$M_{\mathrm{bb}}=\mathrm{diag}\left\{\begin{array}{cccc} M_{b1} & M_{b2} & \cdots & M_{bn_{b}} \end{array}\right\}$$

with

$$M_{bi}=\rho_{b}\int_{0}^{l_{b}}A_{b}(x)N_{u,b}^{T}N_{u,b}dx+\rho_{b}\int_{0}^{l_{b}}A_{b}(x)N_{v,b}^{T}N_{v,b}dx$$

The stiffness submatrix $K_{\mathrm{bb}}$ of the bridge with the order $n_{b}\times n_{b}$ can be expressed by:

(20) $$K_{\mathrm{bb}}=\mathrm{diag}\left\{\begin{array}{cccc} K_{b1} & K_{b2} & \cdots & K_{bn_{b}} \end{array}\right\}$$

with

$$K_{bi}=E_{b}\int_{0}^{l_{b}}A_{b}(x){N^{\prime }}_{v,b}^{T}{N^{\prime }}_{v,b}dx+E_{b}\int_{0}^{l_{b}}I_{b}(x){N^{\prime \prime }}_{v,b}^{T}{N^{\prime \prime }}_{v,b}dx+k_{\mathrm{rb}}\int_{0}^{l_{b}}N_{v,b}^{T}N_{v,b}dx$$

The damping submatrix $C_{\mathrm{bb}}$ of the bridge with the order $n_{b}\times n_{b}$ can be expressed by:

(21) $$C_{\mathrm{bb}}=\mathrm{diag}\left\{\begin{array}{cccc} C_{b1} & C_{b2} & \cdots & C_{bn_{b}} \end{array}\right\}$$

with

$$C_{bi}=c_{b}\int_{0}^{l_{b}}N_{u,b}^{T}N_{u,b}dx+c_{b}\int_{0}^{l_{b}}N_{v,b}^{T}N_{v,b}dx+c_{\mathrm{rb}}\int_{0}^{l_{b}}N_{v,b}^{T}N_{v,b}dx$$

The bridge–rail coupling submatrices are marked with the subscripts “br” and “rb”. The stiffness submatrices $K_{\mathrm{br}}$ with the order $n_{b}\times n_{r}$ and $K_{\mathrm{rb}}$ with the order $n_{r}\times n_{b}$ can be expressed by:

(22) $$K_{\mathrm{br}}=\mathrm{diag}\left\{\begin{array}{cccc} K_{\mathrm{br}1} & K_{\mathrm{br}2} & \cdots & K_{\mathrm{br}n_{b}} \end{array}\right\}$$

(23) $$K_{\mathrm{rb}}=\mathrm{diag}\left\{\begin{array}{cccc} K_{\mathrm{rb}1} & K_{\mathrm{rb}2} & \cdots & K_{\mathrm{br}n_{r}} \end{array}\right\}$$

with

$$K_{\mathrm{br}i}=-k_{\mathrm{rb}}\int_{0}^{l_{b}}N_{v,b}^{T}N_{v,r}dx\;K_{\mathrm{rb}i}=-k_{\mathrm{rb}}\int_{0}^{l_{r}}N_{v,r}^{T}N_{v,b}dx$$

The damping submatrices $C_{\mathrm{br}}$ with the order $n_{b}\times n_{r}$ and $C_{\mathrm{rb}}$ with the order $n_{r}\times n_{b}$ can be expressed by:

(24) $$C_{\mathrm{br}}=\mathrm{diag}\left\{\begin{array}{cccc} C_{\mathrm{br}1} & C_{\mathrm{br}2} & \cdots & C_{\mathrm{br}n_{b}} \end{array}\right\}$$

(25) $$C_{\mathrm{rb}}=\mathrm{diag}\left\{\begin{array}{cccc} C_{\mathrm{rb}1} & C_{\mathrm{rb}2} & \cdots & C_{\mathrm{br}n_{r}} \end{array}\right\}$$

with

$$C_{\mathrm{br}i}=-c_{\mathrm{rb}}\int_{0}^{l_{b}}N_{v,b}^{T}N_{v,r}dx\;C_{\mathrm{rb}i}=-c_{\mathrm{rb}}\int_{0}^{l_{r}}N_{v,r}^{T}N_{v,b}dx$$

### 3.2. Submatrix of Rail

Substituting the displacement fields, i.e., Equations (15)–(18), into the virtual work Equation (4) yields the rail submatrix. The rail submatrix is marked with the subscript “rr”. The mass submatrix $M_{\mathrm{rr}}$ of the rail with the order $n_{r}\times n_{r}$ can be expressed by:

(26) $$M_{\mathrm{rr}}=\mathrm{diag}\left\{\begin{array}{cccc} M_{r1} & M_{r2} & \cdots & M_{rn_{r}} \end{array}\right\}$$

with

$$M_{ri}=\rho_{r}A_{r}\int_{0}^{l_{r}}N_{u,r}^{T}N_{u,r}dx+\rho_{r}A_{r}\int_{0}^{l_{r}}N_{v,r}^{T}N_{v,r}dx$$

The stiffness submatrix $K_{\mathrm{rr}}$ of the rail with the order $n_{r}\times n_{r}$ can be expressed by:

(27) $$K_{\mathrm{rr}}=\mathrm{diag}\left\{\begin{array}{cccc} K_{r1} & K_{r2} & \cdots & K_{rn_{r}} \end{array}\right\}$$

with

$$K_{ri}=E_{r}A_{r}\int_{0}^{l_{r}}{N^{\prime }}_{v,r}^{T}{N^{\prime }}_{v,r}dx+E_{r}I_{r}\int_{0}^{l_{r}}{N^{\prime \prime }}_{v,r}^{T}{N^{\prime \prime }}_{v,r}dx+k_{\mathrm{rb}}\int_{0}^{l_{r}}N_{v,r}^{T}N_{v,r}dx+\sum_{k=0}^{n_{w}}a_{wk}k_{\mathrm{wr}}N_{v,r}^{T}N_{v,r}$$

The damping submatrix $C_{\mathrm{rr}}$ of the rail with the order $n_{r}\times n_{r}$ can be expressed by:

(28) $$C_{\mathrm{rr}}=\mathrm{diag}\left\{\begin{array}{cccc} C_{r1} & C_{r2} & \cdots & C_{rn_{r}} \end{array}\right\}$$

with

$$C_{ri}=c_{\mathrm{rb}}\int_{0}^{l_{r}}N_{v,r}^{T}N_{v,r}dx$$

### 3.3. Submatrices of Heavy-Haul Train and Train–Rail Coupling

The displacement vector of each heavy-haul train comprises six degrees of freedom, which can be expressed by:

(29) $$d_{v}=\left\{\begin{array}{cccccc} v_{c} & \theta_{c} & v_{t1} & \theta_{t1} & v_{t2} & \theta_{t2} \end{array}\right\}$$

The mass matrix $M_{\mathrm{vv}}$, stiffness matrix $K_{\mathrm{vv}}$ and damping matrix $C_{\mathrm{vv}}$ of the heavy-haul train can be derived from the virtual work Equations (5)–(7). The mass matrix $M_{\mathrm{vv}}$ with the order $n_{v}\times n_{v}$ can be expressed by:

(30) $$M_{\mathrm{vv}}=\mathrm{diag}\left\{\begin{array}{cccc} M_{v1} & M_{v2} & \cdots & M_{vn_{v}} \end{array}\right\}$$

with

$$M_{vi}=\mathrm{diag}\left\{\begin{array}{cccccc} m_{c} & I_{c} & m_{t}+2m_{w} & I_{t}+2m_{w}l_{t}^{2} & m_{t}+2m_{w} & I_{t}+2m_{w}l_{t}^{2} \end{array}\right\}$$

The stiffness matrix $K_{\mathrm{vv}}$ with the order $n_{v}\times n_{v}$ can be expressed by:

(31) $$K_{\mathrm{vv}}=\mathrm{diag}\left\{\begin{array}{cccc} K_{v1} & K_{v2} & \cdots & K_{vn_{v}} \end{array}\right\}$$

with

$$\begin{array}{l} K_{vi}=\left[\begin{array}{cccccc} 2k_{2} & 0 & -k_{2} & 0 & -k_{2} & 0\\ & 2k_{2}L_{c}^{2} & -k_{2}L_{c} & 0 & k_{2}L_{c} & 0\\ & & k_{2}+\sum_{k=1}^{2}a_{wk}k_{\mathrm{wr}} & \sum_{k=1}^{2}\eta_{k}a_{wk}k_{\mathrm{wr}}l_{t} & 0 & 0\\ & & & \sum_{k=1}^{2}a_{wk}k_{wr}l_{t}^{2} & 0 & 0\\ & & symm. & & k_{2}+\sum_{k=3}^{4}a_{wk}k_{wr} & \sum_{k=3}^{4}\eta_{k}a_{wk}k_{wr}l_{t}\\ & & & & & \sum_{k=3}^{4}a_{wk}k_{wr}l_{t}^{2} \end{array}\right]\\ \eta_{k}=\left\{\begin{array}{c} 1\;k=1,3\\ -1\;k=2,4 \end{array}\right. \end{array}$$

The damping matrix $C_{\mathrm{vv}}$ with the order $n_{v}\times n_{v}$ can be expressed by:

(32) $$C_{\mathrm{vv}}=\mathrm{diag}\left\{\begin{array}{cccc} C_{v1} & C_{v2} & \cdots & C_{vn_{v}} \end{array}\right\}$$

with

$$C_{vi}=\left[\begin{array}{cccccc} 2c_{2} & 0 & -c_{2} & 0 & -c_{2} & 0\\ & 2c_{2}L_{c}^{2} & -c_{2}L_{c} & 0 & c_{2}L_{c} & 0\\ & & c_{2} & 0 & 0 & 0\\ & & & 0 & 0 & 0\\ & & symm. & & c_{2} & 0\\ & & & & & 0 \end{array}\right]$$

The load vector $F_{v}$ of the train with the order $n_{v}\times 1$, which contains the vector of vehicle gravity $F_{v,g}$ and additional force $F_{v,a}$ induced by track irregularity, can be expressed by:

(33) $$F_{v}=F_{v,g}+F_{v,a}$$

with

$$\begin{array}{l} F_{v,g}={\left\{\begin{array}{cccccccc} 0 & m_{c}g & 0 & (m_{t}+2m_{w})g & 0 & (m_{t}+2m_{w})g & 0 & 0 \end{array}\right\}}^{T}\\ F_{v,a}={\left\{\begin{array}{cccccccc} 0 & 0 & 0 & \sum_{k=1}^{2}a_{wk}k_{\mathrm{wr}}IR_{yk} & \sum_{k=1}^{2}\eta_{k}a_{wk}k_{\mathrm{wr}}l_{t}IR_{yk} & \sum_{k=3}^{4}a_{wk}k_{\mathrm{wr}}IR_{yk} & \sum_{k=3}^{4}\eta_{k}a_{wk}k_{\mathrm{wr}}l_{t}IR_{yk} & 0 \end{array}\right\}}^{T} \end{array}$$

The train–rail coupling submatrices are marked with the subscripts “vr” and “rv”. The stiffness submatrices $K_{\mathrm{vr}}$ with the order $n_{v}\times n_{r}$ and $K_{\mathrm{rv}}$ with the order $n_{r}\times n_{v}$ can be expressed by:

(34) $$K_{\mathrm{vr}}=K_{\mathrm{rv}}^{T}==\mathrm{diag}\left\{\begin{array}{cccc} K_{\mathrm{vr}1} & K_{\mathrm{vr}2} & \cdots & K_{\mathrm{vr}n_{v}} \end{array}\right\}$$

with

$$K_{\mathrm{vr}i}=\left\{\begin{array}{cccccc} 0 & 0 & \sum_{k=1}^{2}a_{wk}k_{\mathrm{vr}}N_{v,r} & \sum_{k=1}^{2}\eta_{k}a_{wk}k_{\mathrm{wr}}l_{t}N_{v,r} & \sum_{k=3}^{4}a_{wk}k_{\mathrm{wr}}N_{v,r} & \sum_{k=3}^{4}\eta_{k}a_{wk}k_{\mathrm{wr}}l_{t}N_{v,r} \end{array}\right\}$$

The load vector $F_{r}$ of the rail induced by track irregularities with the order $n_{r}\times 1$ can be expressed by:

(35) $$F_{r}={\left\{\begin{array}{ccc} 0 & \sum_{k=0}^{n_{w}}a_{wk}k_{\mathrm{wr}}N_{v,r}IR_{yk} & 0 \end{array}\right\}}^{T}(k=1\sim 4)$$

### 3.4. Numerical Integration of Coupling Matrix

The passage of heavy-haul trains through the bridge (track) is discretized. The Newmark-*β* method is employed to solve the train–track–bridge coupling equation at each time step, which assumes that the displacement, velocity and acceleration at previous time $t_{k}$ have been obtained to calculate the current state of motion at $t_{k+1}$. The equation of motion at time $t_{k+1}$ can be expressed by:

(36) $$M{\ddot{X}}_{k+1}+C{\dot{X}}_{k+1}+KX_{k+1}=F_{k+1}$$

By integrating the acceleration from time $t_{k}$ to $t_{k+1}$, the state of current motion at $t_{k+1}$ can be obtained:

(37) $${\ddot{X}}_{k+1}=\frac{1}{\beta \Delta t^{2}}(X_{k+1}-X_{k})-\frac{1}{\beta \Delta t}{\dot{X}}_{k}-\left(\frac{1}{2\beta }-1\right){\ddot{X}}_{k}$$

(38) $${\dot{X}}_{k+1}=\frac{\gamma }{\beta \Delta t}(X_{k+1}-X_{k})-\left(1-\frac{\gamma }{\beta }\right){\dot{X}}_{k}+\left(\frac{\gamma }{2\beta }-1\right){\ddot{X}}_{k}\Delta t$$

(39) $$X_{k+1}={\hat{K}}^{-1}{\hat{F}}_{k+1}$$

with

$$\hat{K}=K+\frac{1}{\beta \Delta t^{2}}M+\frac{1}{\beta \Delta t}C$$

$${\hat{F}}_{k+1}=F_{k+1}+\left[\frac{1}{\beta \Delta t^{2}}X_{k}+\frac{1}{\beta \Delta t}{\dot{X}}_{k}+\left(\frac{1}{2\beta }-1\right){\ddot{X}}_{k}\right]M+\left[\frac{\gamma }{\beta \Delta t}X_{k}+\left(\frac{\gamma }{\beta }-1\right){\dot{X}}_{k}+\frac{\Delta t}{2}\left(\frac{\gamma }{\beta }-2\right){\ddot{X}}_{k}\right]C$$

When *γ* = 1/2 and *β* = 1/4, the Newmark-*β* method is unconditionally stable. The dynamic responses of the heavy-haul train–track–bridge coupling system at discrete time intervals can be solved by Equations (37)–(39).

**Figure 3:**
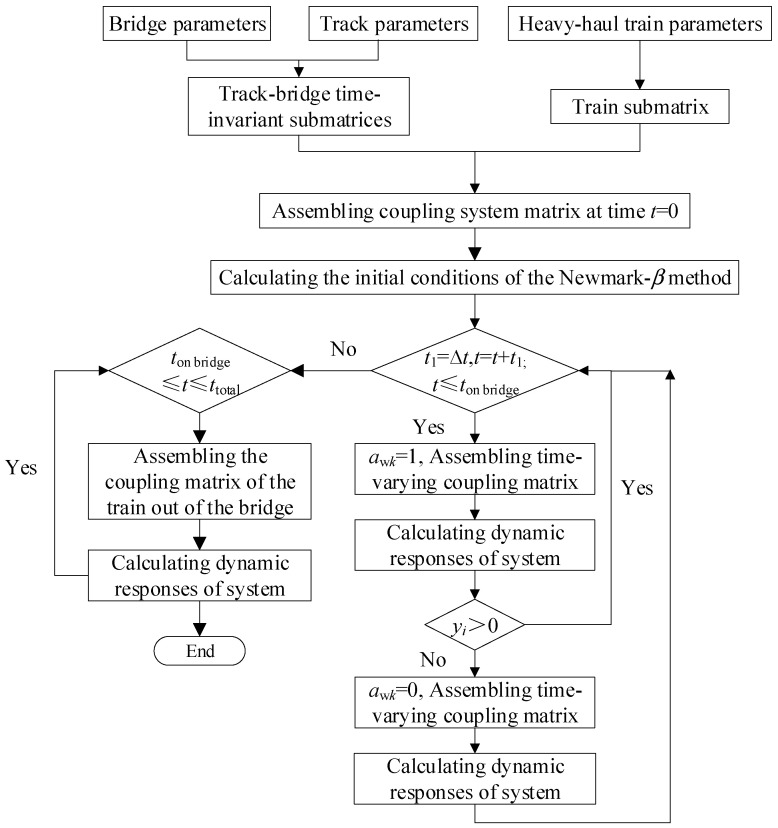
The calculation flow of the heavy-haul train–track–bridge coupling system.

From the above analysis, it can be seen that the coupling between the track and the bridge remains time-invariant, while the coupling between the heavy-haul train and the track exhibits time-varying characteristics. The time-invariant submatrices are initially assembled in the solution, followed by the establishment of the integral heavy-haul train–track–bridge coupling matrix in each integration, taking into account the time step and the varying component of wheel–rail contact stiffness. Finally, the dynamic equations are solved by the Newmark-*β* method. The calculation flow of the heavy-haul train–track–bridge coupling system is shown in Figure 3, and the computational program is compiled by MATLAB programming language.

## 4. Case Study

### 4.1. Project Background

**Figure 4:**
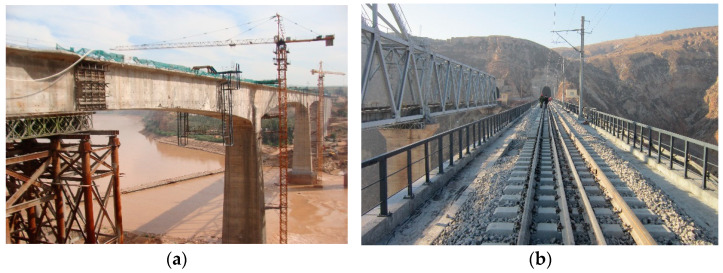
Yellow River Bridge: (**a**) photograph; (**b**) track structure on bridge.

The Yellow River Bridge, located on a heavy-haul railway line, is the subject of investigation, as shown in Figure 4. The bridge is a single-track structure designed for a speed of 80 km/h, with a total length of 325.4 m. The bridge structure is a continuous rigid frame bridge featuring a single-hole box girder with a variable cross-section. The spans of the left, middle and right are 96.7 m, 132.0 m and 96.7 m, respectively. The ballasted track is laid on the bridge with a rail mass of 60 kg/m per unit length.

**Figure 5:**
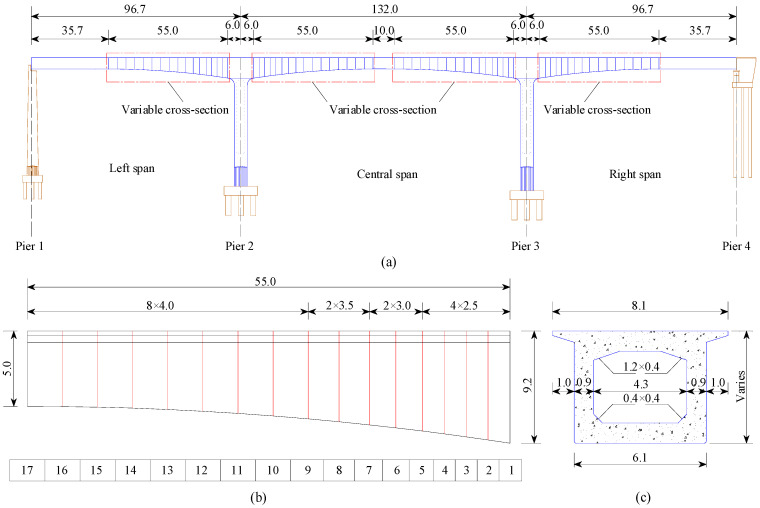
Yellow River Bridge model (unit: m): (**a**) side view; (**b**) key section number of variable cross-section; (**c**) cross-section.

The side view model of the bridge is depicted in Figure 5a. There are four sections of variable cross-section with parabolic heights, each 55.0 m in length on both sides of Piers 2 and 3, as shown in Figure 5b. The variable section of the bridge is divided into 16 elements at unequal intervals based on the length of each construction section. The main beam consists of 17 key sections. The cross-section is shown in Figure 5c. The inertia moment and area parameters for 17 key sections are presented in Table 1.

In order to effectively release the axial displacement of the bridge, a consolidation mode is adopted at the pier–beam connection, and the other two piers are provided with supports. The box girder is constructed using C55 concrete, which has a density of 26.5 kN/m^3^ and an elastic modulus of 35.5 GPa. The pier body is constructed using C45 concrete, which has an elastic modulus of 34.5 GPa and a Poisson’s ratio of 0.2.

The following parameters are assumed for the track: $E_{r}=2.06\times 10^{11}\mathrm{Pa}$, $I_{r}=3.217\times 10^{-5}m^{4}$, $m_{r}=60.64\;\mathrm{kg}/m$, $k_{\mathrm{rb}}=6\times 10^{7}N/m$ and $c_{\mathrm{rb}}=7.5\times 10^{4}N\cdot s/m$. In addition, the following parameters are assumed for the heavy-haul train: $m_{c}=9\times 10^{4}\mathrm{kg}$, $I_{c}=1.4075\times 10^{6}\mathrm{kg}\cdot m^{2}$, $m_{t}=460\;\mathrm{kg}$, $I_{t}=175\;\mathrm{kg}\cdot m^{2}$, $L_{c}=4.6\;m$, $l_{t}=0.915\;m$, $L_{v}=12\;m$ (total length of each vehicle), $k_{2}=1.9\times 10^{6}N/m$, $c_{2}=3\times 10^{6}N\cdot s/m$ and $k_{\mathrm{wr}}=2.8\times 10^{8}N/m$.

### 4.2. Dynamic Analysis on the Effect of Track Irregularities

The presence of geometric irregularities on the track is a significant factor contributing to the excitation of vibration in the heavy-haul train–track–bridge coupling system. The wavelength range of track irregularities is extensive, encompassing numerous harmonic components with varying amplitudes and wavelengths. The common wavelength range of the track irregularity is 0.01~200 m, in which the short and medium wave have great effects on the vibration of the coupling system. In order to comprehensively capture the vibrations of trains, tracks, and bridges caused by track geometries, track irregularities of different wavelengths must be included in the dynamic analysis.

**Figure 6:**
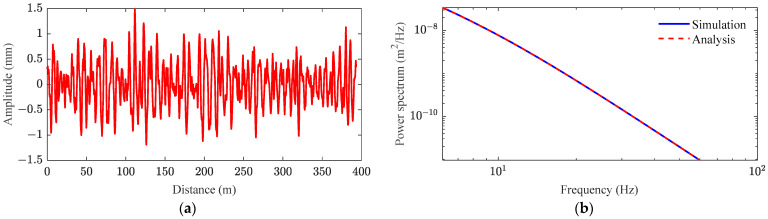
Time–frequency transform of track irregularity spectrum: (**a**) sample; (**b**) comparison between simulation and analysis.

The heavy-haul train–track–bridge coupling dynamic model, based on the virtual work principle, incorporates a time domain input as the system excitation. Inverse Fourier transform (IFFT) is used to transform the power spectral density function of the track’s random irregularity into a spatial sample that varies with the distance traveled along the track. The low interference spectrum is used to generate longitudinal irregularity with a wavelength range of 1 to 10 m, as shown in Figure 6. It can be seen that the simulation of the power spectral density is consistent with the analysis, indicating that the generated track irregularity sample is reasonable and effective.

**Figure 7:**
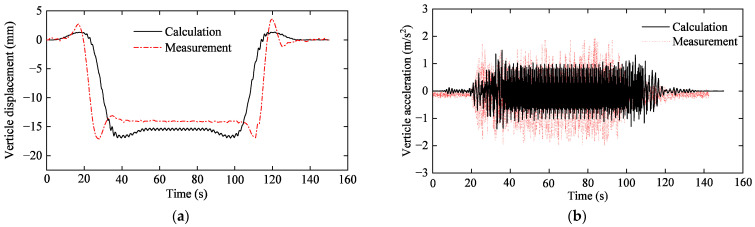
Comparison of mid-span measurement and calculation: (**a**) vertical displacement; (**b**) vertical acceleration.

To better verify the effectiveness of the proposed model, a vibration test of the middle span of the Yellow River Bridge was carried out. The displacement and acceleration obtained from the proposed model and the experimental test are compared in Figure 7. It can be seen that the calculated displacement and acceleration are consistent with the measured results in terms of trend. Among them, the calculated absolute maximum displacement and acceleration of the bridge are 16.96 mm and 1.56 m/s^2^, respectively, while they are 17.13 mm and 1.95 m/s^2^ in the field test. The discrepancy of displacement in amplitudes is limited, ranging from 1% to 11%. However, there exists a slightly larger disparity between the simulated and measured acceleration. One reason may be that the track irregularities in other wavelength ranges are sensitive to bridge acceleration. Other primary factors contributing to the inconsistency include the inconsistent axle loads, structural fatigue damages, uncertain measurement noises and environmental changes.

**Figure 8:**
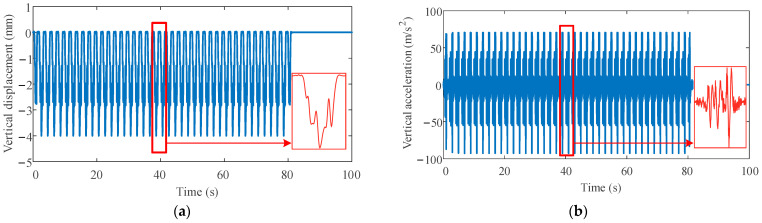
Dynamic responses of the rail in the transition section (red box contains local amplification): (**a**) vertical displacement; (**b**) vertical acceleration.

The vertical displacement and acceleration of the rail in the transition section are shown in Figure 8. Ballast can be clearly observed to effectively attenuate the free vibration caused by axle loads of the heavy-haul train. The heavy-haul train lacking primary suspension fails to effectively mitigate the impact of the nodding motion of the bogie on the track.

**Figure 9:**
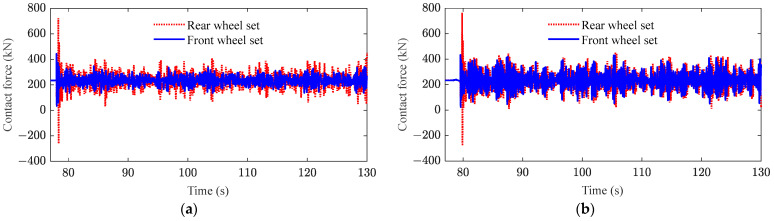
Wheel–rail contact force: (**a**) front bogie; (**b**) rear bogie.

Figure 9 shows the contact force between the front wheelset and the rear wheelsets of both the front and rear bogies of a vehicle, from which one can notice that the variation in wheelset contact force is more violent in the rear bogie compared to the front bogie. The fluctuation of contact force of the rear wheelset significantly increases, indicating that the nodding motion of the front bogie has a significant effect on the contact force of the rear wheelset.

**Figure 10:**
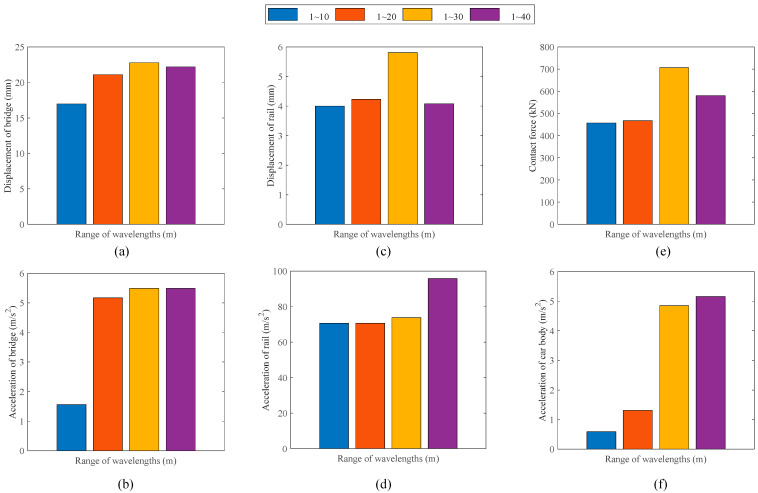
Maximum dynamic response in different wavelength ranges: (**a**) mid-span displacement of bridge; (**b**) mid-span acceleration of bridge; (**c**) track displacement in transition section; (**d**) track acceleration in transition section; (**e**) wheel–rail contact force; (**f**) acceleration of car body.

The maximum dynamic responses of the system in the four wavelength ranges, i.e., 1~10 m, 1~20 m, 1~30 m and 1~40 m, are shown in Figure 10. The track irregularities in the wavelength range of 1 to 20 m have a significant effect on the responses of the bridge, whereas wavelengths greater than 20 m show a negligible influence on the vibration of the bridge. The resonance of the car body occurs when the wavelength exceeds 20 m, resulting in a substantial amplification of both the acceleration amplitude of the car body and the wheel–rail contact force. The influence of the interaction between the heavy-haul train and the rail on the track vibration is significantly more pronounced compared to that of the interaction between the bridge and the track.

### 4.3. Dynamic Analysis on the Effect of Axle Load

Increasing the axle loads of a heavy-haul train can significantly improve the efficiency of transportation. In order to investigate the influence of axle load on the dynamic behavior of system, three kinds of axle loads of 25 t, 27.5 t and 30 t are studied. In this section, calculations still consider track irregularities with wavelengths ranging from 1 to 10 m.

**Figure 11:**
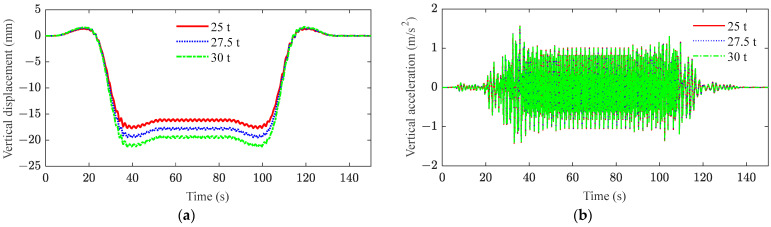
Mid-span responses of the bridge under different axle loads: (**a**) vertical displacement; (**b**) vertical acceleration.

**Figure 12:**
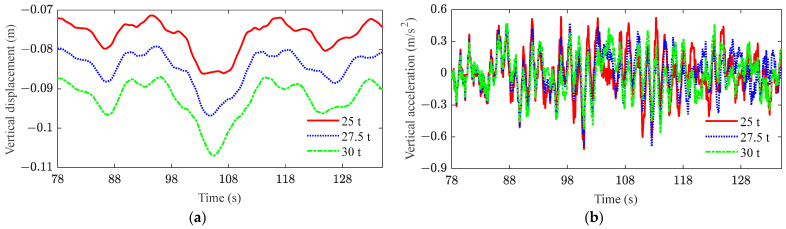
Responses of car body under different axle loads: (**a**) vertical displacement; (**b**) vertical acceleration.

Figure 11 and Figure 12 show the effects of mid-span displacement and acceleration of the bridge, as well as car body displacement and acceleration under track irregular excitation, respectively. From these results, it can be seen that the displacement response of the system increases linearly with the increase in axle load. When the freight volume of heavy-haul trains increases, the displacement can be reduced by strengthening measures to improve the stiffness of the structure. In Figure 11b, the increase of axle load has a minimal impact on bridge acceleration. The coupling vibration and the irregular excitation of the track are the main factors affecting bridge acceleration. As shown in Figure 12b, the increase in the mass of the car body can restrain the peak acceleration of the car body to a certain extent. The irregular track excitation has a significant effect on the acceleration of the car body if the freight volume of the vehicle is small. The utilization of a new heavy-haul train equipped with primary suspension can effectively enhance the vibration isolation performance against track irregular excitation, thereby mitigating the acceleration of the car body.

### 4.4. Dynamic Analysis on the Effect of Bridge Structural Degradation

Under the cyclic loading of heavy-haul trains, structural fatigue and performance degradation of railway bridges are inevitable. Rayleigh damping is adopted into the bridge model. Considering track irregularities with wavelengths ranging from 1 to 10 m as excitation, a degradation of overall stiffness and damping by 10% and 20%, respectively, is taken into account.

**Figure 13:**
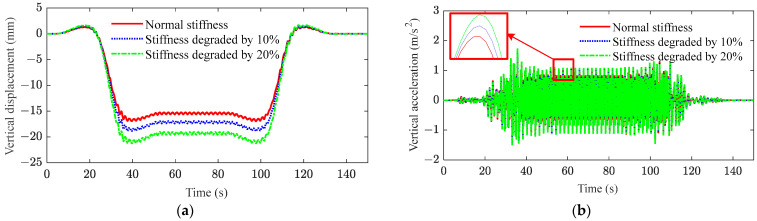
Mid-span responses of bridge under stiffness degradation: (**a**) vertical displacement; (**b**) vertical acceleration (red box contains local amplification).

**Figure 14:**
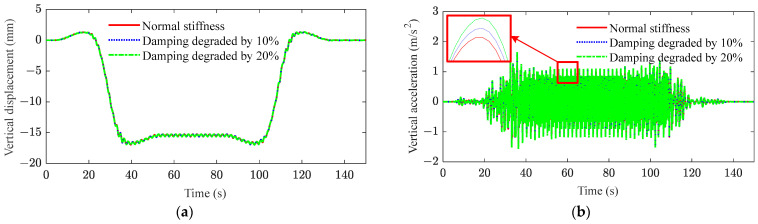
Mid-span responses of bridge under damping degradation: (**a**) vertical displacement; (**b**) vertical acceleration (red box contains local amplification).

Figure 13 and Figure 14 plot the responses of the displacement and acceleration in the middle span of the bridge after the stiffness and damping of the bridge are degraded, respectively. From these results, it can be observed that the stiffness degradation of the bridge has the most significant influence on the displacement of the bridge. As shown in Figure 13a and Figure 14a, the stiffness of the bridge is reduced by 10% and 20%, respectively, while the mid-span displacement of the bridge is increased by 11% and 25%, respectively. However, the decrease in bridge damping can be disregarded when considering the displacement in the middle of the bridge span. As shown in Figure 13b and Figure 14b, the decrease in bridge stiffness and damping leads to an increase in the amplitude of acceleration. However, the magnitude of the change is not obvious. Therefore, the fatigue cracking of the bridge occurs due to the cyclic loading from heavy-haul trains, resulting in a reduction in bridge stiffness. The excessive deformation further deteriorates the mechanical performance of the bridge and exacerbates its dynamic behavior.

## 5. Conclusions

A heavy-haul train–track–bridge coupling system, based on the virtual work principle and represented by a coupling matrix, is presented in this paper. The research focuses on a continuous rigid frame bridge accommodating a heavy-haul railway line as the object of study. The validity and accuracy of the proposed model are verified through comparison with the model and experimental results. The following major conclusions are drawn from this study:

(a) According to the vibration characteristics of the interaction between the heavy-haul train, track and bridge, the motion equation of the train–track–bridge coupling system is derived by the principle of virtual work, which is combined with the displacement field to obtain the expression of the coupling matrix of the motion equation of the whole system. The dynamic responses of the heavy-haul train, track and bridge are solved by a step-by-step integration method at the same time;
(b) Notably, the contact force fluctuations on the rear wheelset of heavy-haul trains are considerably higher than those on the front wheelset within the same bogie. This discrepancy arises due to the absence of primary suspension isolating the nodding motion of the bogie. Additionally, the bridge’s vibration is particularly sensitive to track irregularities with wavelengths ranging from 1 to 20 m, especially within the 1 to 10 m range;
(c) The displacement of both the bridge and the car body increases linearly as the load on the car body increases, while the acceleration of the bridge and car body have negligible influence;
(d) The study incorporates the impact of performance degradation on bridge vibration by reducing the bridge’s stiffness and damping by 10% and 20%, respectively. This attenuation significantly affects the vertical displacement of the bridge, whereas the impact on vertical displacement due to damping attenuation can be disregarded. Both the stiffness and damping attenuation contribute to a subtle increase in the bridge’s acceleration.

## Figures and Tables

**Table 1:** Parameters of the key sections of Yellow River Bridge.

Section	1	2	3	4	5	6	7	8	9
Area (m^2^)	23.92	22.74	21.95	21.19	20.48	15.96	15.38	14.76	14.20
Inertia moment (m^4^)	247.47	210.91	188.35	168.38	150.74	120.48	106.24	92.18	80.50
Section	10	11	12	13	14	15	16	17	
Area (m^2^)	13.62	13.13	12.71	12.36	12.09	10.65	10.55	10.52	
Inertia moment (m^4^)	69.61	60.95	54.17	48.98	45.16	40.60	39.14	38.66	

## Data Availability

Not applicable.
